# Supplementary material for: Multidimensional gender discrimination in workplace and depressive symptoms
Source: PLoS One. 2020 Jul 16;15(7):e0234415. doi: 10.1371/journal.pone.0234415 (PMC7365387; doi:10.1371/journal.pone.0234415)
Supplement: Attachment — Submitted filename: response to reviewers.docx [file pone.0234415.s001.docx]

Multidimensional Gender Discrimination in Workplace and Depressive Symptoms

**General Response**

Dear reviewers and editor:

We are grateful for the insightful comments from you. We have edited our manuscript according to reviewers’ comments point by point. We would like to express my sincere gratitude to you. Thank you.

Reviewer Comments:
Reviewer 1
The authors investigate an important topic on gender discrimination. Additionally, the large sample size with a good sampling method increase the external validity of the study findings. However, some revisions are needed before I recommend publication. Please see my comments below.

Comment 1). For the Introduction, please describe more thoroughly in the concept of discrimination and stigma. Specifically, I would like to see the authors mention that different populations (e.g., people with mental illness, people with overweight, people who are transgender, people who have same-sex marriage) all face the difficulties of discrimination. Then, the authors can narrow down the discrimination problem from different populations to their study topic; that is, female.
There is an ongoing special issue in the IJERPH, which collect essential information for the authors to consider and cite: <https://www.mdpi.com/journal/ijerph/special_issues/stigma>
The authors may also consider the following reference if relevant:
Ahorsu, D. K., Lin, C.-Y., Imani, V., Griffiths, M. D., Su, J.-A., Latner, J. D., Marshall, R. D., Pakpour, A. H. (2020). A prospective study on the link between weight-related self-stigma and binge eating: Role of food addiction and psychological distress. International Journal of Eating Disorders, 53(3), 442-450.
Alimoradi, Z., Golboni, F., Griffiths, M. D., Broström, A., Lin, C.-Y., & Pakpour, A. H. (in press). Weight-related stigma and psychological distress: A systematic review and meta-analysis. Clinical Nutrition. <https://doi.org/10.1016/j.clnu.2019.10.016>
Lin, C.-Y., Imani, V., Broström, A., Huus, K., Björk, M., Hodges, E. A., Pakpour, A. H. (in press). Psychological distress and quality of life in Iranian adolescents with overweight/obesity: Mediating role of weight bias internalization and insomnia. Eating and Weight Disorders. doi: 10.1007/s40519-019-00795-5

**(Response) Thank you for your constructive comments. We have added the concept of stigma and examples of discrimination in different populations.**

**In introduction section,**

**“**When a group of people is stigmatized based on their characteristic, since stigma is linked to social difference, they could easily face the difficulties of discrimination. The population can be minority immigrant, lesbian, gay, bisexual, transgender, and Queer (LGBTQ), people who are overweight, people with health problems (e.g. AIDS or mental illness), or female gender. Discrimination is found in wide situations, such as social isolation of minority immigrant youth[1], experience of being bullied in adolescents with obesity[2], housing discrimination based on sexual orientation[3], or employment discrimination against people with AIDS[4].”, and

“[5]. Among people who had experienced discrimination due to their HIV status, internalized stigma significantly predicted cognitive-affective depression[6]. Weight-related perceived stigma as well as self-stigma is shown to be associated with psychological distress[7]. Recent studies have also shown that gender-based discrimination also produce deleterious health impacts such as cardiovascular disease [8], may increase drinking and smoking behaviors [9], and can aggravate depressive symptoms [10].”

Please read our revised manuscript, too.

Comments 2). For the first sentence in the Introduction, the authors mentioned the high prevalence of depression worldwide. Please directly provide the prevalence for readers to have a better idea here.

**(Response) We added sentence regarding prevalence of depression, as your comment.**

“The 12-month prevalence estimate of major depressive episodes averaged 3.2% in healthy participants and 9.3% to 23.0% in participants with comorbid physical disease in the WHO World Health Survey across 60 countries[11].”

Commnet 3). A major problem in the present study is that it is unclear how the gender discrimination was assessed. The authors mentioned "Participants were specifically asked whether they had experienced any of these types [hiring, promotion, paid wages, work assignments, training opportunities, and firing] according to a response scale for each item ranging from “never,” “rarely,” “almost,” to “always.”" The problem here is that only reading the descriptions, I cannot observe any "discrimination" concept here. For example, if the question is "Do you have any problems in hiring?" Then, this example question is not at all "discrimination". Therefore, the authors should give detailed information on the discrimination measure, especially this is their study's core concept.

**(Response) The exact questionnaires were as follows. And we added the sentence below in methods section. Please read our revised manuscript, too.**

In method section,

(1) Hiring: If candidates have similar qualifications for appointment, men are preferred to women.

(2) Promotion: Even with identical or similar careers, male workers are promoted faster than female counterparts.

(3) Paid wages: Even in identical or similar positions, male workers receive higher wages and bonuses than female workers.

(4) Work assignments: Duties are fixed or customarily divided between men and women.

(5) Training opportunities: Even with similar duties, men have more opportunities of receiving education and training than women.

(6) Firing: In case of restructuring, female workers are more likely to be forced to quit.

4. Please provide psychometric properties of the short version CES-D, especially how the sensitivity and specificity of the cutoff 10 in the CES-D.

**(Response) Thank you for your comments. The CES-D is wide used psychological questionnaires. And we reviewed the article about psychometric properties of CESD, and summarized it into method section, as below. Furthermore, we added validation (such as Kappa value, sensitivity, specificity) in method section.**

“CES-D 10 has been proven as a reliable alternative to the original CES-D 20(Kappa=0.82, P<0.001) in classifying participants with depressive symptoms (sensitivity 91%, specificity 92%, positive predictive values 92%)”

5. It is unclear whether all the discrimination items (hiring, promotion, paid wages, work assignments, training opportunities, and firing) were included in the same Model 2 of logistic regression model.

(Response) One of discrimination item was included in each logistic regression model (Crude). So we repeated 6 times to make Crude OR column of Table 3. Then, we adjusted variables for each logistic regression model.

6. Following my comment #5, please use AOR instead of OR for Model 2; and COR instead of OR for the Crude Model. Also, add the footnotes to explain AOR (adjusted odds ratio) and COR (crude odds ratio).

(Response 6) Thank you for your detailed comment. we used full name of each OR as “crude odds ratio” and “adjusted odds ratio” in row of Table as your comment.

7. Page 14, "The government established the Sex Discrimination Act in 2005....." should be explicitly indicate that it is South Korean government.

(Response 7) We added the ‘South Korean government’, in that sentence.

8. Apart from the limitation of self-report, I think that the questionnaire on gender discrimination is not a standardized instrument. Therefore, this should also be acknowledged as a limitation.

(Response 8) We agree that the questionnaire was not validate or standardized for study. So, we added sentences in limitation section as below.

“Also, the questionnaires of current study are not validated or standardized instrument, so they did not measure objective levels of discrimination such as frequency, thereby current study results are not free from systemic error.”

1.	Cherng, H., K. Turney, and G. Kao, *Less socially engaged? Participation in friendship and extracurricular activities among racial/ethnic minority and immigrant adolescents.* Teachers College Record, 2014. **116**(3): p. 1-28.

2.	Lin, Y.C., et al., *Poor health and experiences of being bullied in adolescents: Self‐perceived overweight and frustration with appearance matter.* Obesity, 2018. **26**(2): p. 397-404.

3.	Kattari, S.K., et al., *Policing gender through housing and employment discrimination: comparison of discrimination experiences of transgender and cisgender LGBQ individuals.* Journal of the Society for Social Work and Research, 2016. **7**(3): p. 427-447.

4.	Leonard, A.S., *Employment discrimination against persons with AIDS.* Clearinghouse Rev., 1985. **19**: p. 1292.

5.	Kim, S.S. and D.R. Williams, *Perceived discrimination and self-rated health in South Korea: a nationally representative survey.* PLoS One, 2012. **7**(1): p. e30501.

6.	Simbayi, L.C., et al., *Internalized stigma, discrimination, and depression among men and women living with HIV/AIDS in Cape Town, South Africa.* Social science & medicine, 2007. **64**(9): p. 1823-1831.

7.	Alimoradi, Z., et al., *Weight-related stigma and psychological distress: A systematic review and meta-analysis.* Clinical Nutrition, 2019.

8.	Lewis, T.T., et al., *Self-reported experiences of discrimination and cardiovascular disease.* Current cardiovascular risk reports, 2014. **8**(1): p. 365.

9.	Zucker, A.N. and L.J. Landry, *Embodied discrimination: The relation of sexism and distress to women’s drinking and smoking behaviors.* Sex Roles, 2007. **56**(3-4): p. 193-203.

10.	Pavalko, E.K., K.N. Mossakowski, and V.J. Hamilton, *Does perceived discrimination affect health? Longitudinal relationships between work discrimination and women's physical and emotional health.* Journal of Health and social Behavior, 2003: p. 18-33.

11.	Kessler, R.C. and E.J. Bromet, *The epidemiology of depression across cultures.* Annual review of public health, 2013. **34**: p. 119-138.

12.	Zhang, W., et al., *Validating a shortened depression scale (10 item CES-D) among HIV-positive people in British Columbia, Canada.* PloS one, 2012. **7**(7): p. e40793.
